# FOXO4-Knockdown Suppresses Oxidative Stress-Induced Apoptosis of Early Pro-Angiogenic Cells and Augments Their Neovascularization Capacities in Ischemic Limbs

**DOI:** 10.1371/journal.pone.0092626

**Published:** 2014-03-24

**Authors:** Takaharu Nakayoshi, Ken-ichiro Sasaki, Hidemi Kajimoto, Hiroshi Koiwaya, Masanori Ohtsuka, Takafumi Ueno, Hidetoshi Chibana, Naoki Itaya, Masahiro Sasaki, Shinji Yokoyama, Yoshihiro Fukumoto, Tsutomu Imaizumi

**Affiliations:** 1 Division of Cardio-Vascular Medicine, Department of Internal Medicine, Kurume University School of Medicine, Fukuoka, Japan; 2 The Cardiovascular Research Institute, Kurume University, Fukuoka, Japan; 3 International University of Health and Welfare, Fukuoka, Japan; UAE University, Faculty of Medicine & Health Sciences, United Arab Emirates

## Abstract

The effects of therapeutic angiogenesis by intramuscular injection of early pro-angiogenic cells (EPCs) to ischemic limbs are unsatisfactory. Oxidative stress in the ischemic limbs may accelerate apoptosis of injected EPCs, leading to less neovascularization. Forkhead transcription factor 4 (FOXO4) was reported to play a pivotal role in apoptosis signaling of EPCs in response to oxidative stress. Accordingly, we assessed whether FOXO4-knockdown EPCs (FOXO4^KD^-EPCs) could suppress the oxidative stress-induced apoptosis and augment the neovascularization capacity in ischemic limbs. We transfected small interfering RNA targeted against FOXO4 of human EPCs to generate FOXO4^KD^-EPCs and confirmed a successful knockdown. FOXO4^KD^-EPCs gained resistance to apoptosis in response to hydrogen peroxide *in vitro*. Oxidative stress stained by dihydroethidium was stronger for the immunodeficient rat ischemic limb tissue than for the rat non-ischemic one. Although the number of apoptotic EPCs injected into the rat ischemic limb was greater than that of apoptotic EPCs injected into the rat non-ischemic limb, FOXO4^KD^-EPCs injected into the rat ischemic limb brought less apoptosis and more neovascularization than EPCs. Taken together, the use of FOXO4^KD^-EPCs with resistance to oxidative stress-induced apoptosis may be a new strategy to augment the effects of therapeutic angiogenesis by intramuscular injection of EPCs.

## Introduction

Cell therapy for augmenting neovascularization may be a promising strategy to rescue tissues with ischemia [1]. Early pro-angiogenic cells (EPCs) [2], which were reported to contribute to adult neovascularization, may offer more options for cell therapy. EPCs can be *ex vivo*-expanded out of isolated bone marrow-derived mononuclear cells (BM-MNCs) and peripheral blood-derived mononuclear cells (PB-MNCs) [3], and these cells have been used for cell therapy in large-scale clinical trials [4]. We and others performed therapeutic angiogenesis by intramuscular injection of BM-MNCs and PB-MNCs for patients with critical limb ischemia and reported the effects and safeties [5]–[8]. However, the effects have been unsatisfactory. Although we and others reported that the impaired migration capacity of atherosclerotic patient-derived BM-MNCs and EPCs *in vitro* was related to the impaired neovascularization capacity of the cells *in vivo* [9], [10], there may be other reasons for the unsatisfactory effects.

In ischemic limbs, exposure to acute or chronic hypoxia induces oxidative stress to the skeletal muscle in animals and humans [11]–[13]. In addition, it has been reported that oxidative stress is a mediator of apoptosis [14], [15]. Thus, ischemia-induced oxidative stress may be increased in the limbs of patients with critical limb ischemia and the ischemia-induced oxidative stress may accelerate apoptosis of EPCs injected to the ischemic limbs. The loss of viable EPCs may result in impaired neovascularization of the ischemic limb. If we can augment the anti-apoptosis capacity of EPCs to oxidative stress before the injection, the effects of therapeutic angiogenesis may be more desirable. Therefore, we tried in this study to find out how we could augment the anti-apoptosis capacity of EPCs in response to oxidative stress. Brunet et al. reported that apoptosis of human epithelial cells was suppressed by inactivation of forkhead transcription factor (FOXO) [16]. Nemoto et al. reported that FOXO was activated by reactive oxygen species (ROS) [17]. In EPCs, Urbich et al. reported that FOXO4, but not FOXO1 and FOXO3a, played a pivotal role in a signaling of oxidative stress-induced apoptosis [18]. Accordingly, we transfected small interfering RNA (siRNA) targeted against FOXO4 of EPCs to generate FOXO4-knockdown EPCs (FOXO4^KD^-EPCs) and examined whether FOXO4^KD^-EPCs gained resistance to apoptosis in response to oxidative stress *in vitro*. In addition, we assessed whether intramuscular injection of FOXO4^KD^-EPCs to the rat ischemic limbs could augment neovascularization though the augmented anti-apoptosis capacity to ischemia-induced oxidative stress.

## Materials and Methods

### EPC Culture

We first obtained written informed consent by documentation for this study and then collected peripheral blood from healthy volunteers and coronary artery disease patients with more than two of the following atherosclerotic risk factors: age >65 years, current smoking, hypertension, dyslipidemia, and diabetes. This study conformed to the principles outlined in the Declaration of Helsinki and was approved by the Committees on the Ethics Review Board of Kurume University School of Medicine. We generated EPCs from peripheral blood-derived mononuclear cells (PB-MNCs), as we and others previously reported [9], [19]. In brief, PB-MNCs were isolated by density gradient centrifugation with Ficoll (Ficoll-Paque PLUS, GE Healthcare Bio-Sciences AB) and seeded onto a plate coated with fibronectin (Sigma) and cultured in endothelial basal medium with supplements (EBM-2 BulletKit, Clonetics) and 20% fatal bovine serum (FBS) for 4 days at 37°C, 5% CO_2_, in a humidified incubator.

### EPC Apoptosis Assay

We assessed apoptosis of EPCs *in vitro* and *in vivo* using the terminal dUTP nick-end labeling (TUNEL) method. First, we assessed apoptosis of EPCs subjected to malnutrition stress, hypoxic stress, or oxidative stress *in vitro*. We incubated EPCs with serum-free culture medium, with hypoxic chamber (O_2_ 1.0%; CO_2_ 5.0%, N_2_; 94%; APM-30DR, ASTEC), or with 1 mM hydrogen peroxide (H_2_O_2_)-added culture medium for 18 h at 37°C. H_2_O_2_ was used experimentally as the chemical agent of exogenous oxidative stress [18]. The cells were detached from the culture plates, washed twice with PBS, and fixed with 1% paraformaldehyde. TUNEL-positive cells were stained with an apoptosis detection kit (*In Situ* Cell Death Detection Kit, Fluorescein, Roche) and observed under a fluorescence microscope (BX50, OLYMPUS). In addition, another apoptosis detection kit (APO-DIRECTTM Kit, BD Biosciences) was used to analyze TUNEL-positive cells using flow cytometry (FACSCanto II, Becton Dickinson). These staining procedures were performed according to the respective manufacturers’ instructions. The flow cytometric analysis was performed with software supplied by the manufacturer (FACSDiva, Becton Dickinson). Second, we assessed apoptosis of EPCs injected into the rat ischemic limbs. We labeled EPCs with a fluorescent red reagent Dil (CellTracker CM-Dil C7000, Invitrogen) according to the manufacturer’s instructions and injected a total of 50 μL phosphate buffered saline (PBS) with or without EPCs (5×10^5^ cells per rat) into five equally-spaced points on the ischemic and non-ischemic adductor muscles 2 h after the surgery of hindlimb ischemia. We harvested the tissues of the ischemic and non-ischemic adductor muscles 24 h after the injection of EPCs and fixed 5-μm frozen sections of the tissues with 2% paraformaldehyde for 10 min at room temperature. TUNEL-positive cells in the tissues were stained with an apoptosis detection kit (*In Situ* Cell Death Detection Kit, Fluorescein, Roche) according to the manufacturer’s instructions. We counted the numbers of TUNEL-positive and TUNEL-negative EPCs in 10 randomly selected high-power fields (×400) with a fluorescence microscope (BX50, OLYMPUS) and divided the number of TUNEL-positive EPCs by the total number of EPCs to calculate the apoptosis ratio for the injected EPCs.

### Western Blotting

We harvested EPCs and thigh tissues from the culture plate and rat ischemic limbs, respectively. Then, we lysed them with a buffer (0.5 mM HEPES, pH 7.4, 5.0 M NaCl, 10% Triton X-100, 10% glycerol, 0.2 M Na3VO4, 0.5 M NaF, 0.1 M NaPP). Twenty micrograms of protein per sample was electrophoresed on a polyacrylamide gel (NuPAGE Novex 4–12% Bis-Tris Gel, Invitrogen), and the protein was transferred onto a polyvinylidene difluoride membrane (iBlot Gel Transfer Stacks, Invitrogen). The membrane was blocked with a buffer (Blocking One, Nacalai Tesque, Inc.) and incubated for 24 h at 4°C with primary antibodies as follows: a rabbit anti-human β-actin antibody, a rabbit anti-human FOXO3a antibody, a rabbit anti-human FOXO4 antibody, a rabbit anti-human Bim antibody, a rabbit anti-human cleaved caspase-3 antibody, a rabbit anti-human vascular endothelial growth factor (VEGF) antibody, a rabbit anti-human basic-fibroblast growth factor (b-FGF) antibody (above antibodies were purchased from Cell Signaling Technology), a rabbit anti-human stromal cell-derived factor-1 (SDF-1) antibody, and a rabbit anti-human insulin-like growth factor-1 (IGF-1) antibody (above antibodies were purchased from abcam). The membrane was then washed twice with TBS-Tween and additionally incubated with a corresponding horseradish peroxidase-conjugated secondary antibody (Invitrogen) for 60 min at room temperature. The antigen-antibody complex signal was optically detected using an enhanced chemiluminescence system (SuperSignal West Pico Chemiluminescent Substrate, Thermo Scientific), and the signal density was determined with image analysis computer software (Multi Gauge v3.0, Fuji Film). β-actin or GAPDH was used as a control protein. Calculated densities were expressed as FOXO3a/β-actin, FOXO4/β-actin, Bim/β-actin, cleaved caspase-3/β-actin, VEGF/GAPDH, b-FGF/GAPDH, SDF-1/GAPDH, and IGF-1/GAPDH density ratios.

### siRNA Transfection of EPCs

We detached EPCs from the fibronectin-coated culture plate with trypsin (TrypLE Express, Invitrogen), re-suspended them with the serum-free medium, and re-seeded them onto a plate coated with carboxyl (BD Pure Coat Carboxyl, BD Biosciences) to avoid mixing of fibronectin into the sample of EPCs for western blot analysis. The siRNA transfection of EPCs with either negative control siRNA (sequence: sense 5′-UAACGACGCGACGACGUAAtt, antisense UUACGUCGUCGCGUCGUUAtt-3′) or FOXO4 siRNA (sequence: sense 5′-CCGCGAUCAUAGACCUAGAtt, antisense UCUAGGUCUAUGAUCGCGGca-3′) (Silencer Select siRNA, Applied Biosystems) was performed using the magnetofection method [19], [20].

### EPC Phenotype Assay

We assessed the phenotype of EPCs by flow cytometric analysis. After detaching of EPCs from the culture plate, we incubated the cells with antibodies to the following cell surface antigens: CD133 (PE-labeled, Miltenyi Biotec GmbH), CD34, CD31 (PE-labeled, BD Biosciences), and KDR (Biotin conjugated, Sigma; SAv-FITC conjugated, BD Biosciences). The expressions of the antigens were analyzed by a flow cytometer (FACSCanto II, Becton Dickinson) [9], [19].

### Hindlimb Ischemia Animal Model

We assessed oxidative stress and neovascularization of ischemic limbs using a unilateral hindlimb ischemia animal model with 8-to-12-week-old athymic nude rats (F344/N-rnu/rnu, CLEA Japan Inc.) [19]. The proximal portion of the femoral artery and the distal portion of the saphenous artery were occluded using an electrical coagulator (Vetroson V-10 bi-polar electrosurgical unit, Summit Hill Laboratories), and the overlying skin was closed using nylon sutures. The investigation conformed to the *Guide for the Care and Use of Laboratory Animals* published by the US National Institutes of Health. The study protocol was approved by the Institutional Animal Care and use Committee of Kurume University School of Medicine. All procedures for rats were performed under anesthesia with isoflurane (5% in 100% oxygen for induction, 1–2% in 100% oxygen for maintenance) using an animal anesthesia apparatus (TK-5, Bio Machinery). Depth of anesthesia was monitored by the toe pinch reflex test.

### ROS Production Assay

To assess the oxidative stress of rat ischemic limbs, we analyzed the generation of ROS in the limbs. At 14 days after the hindlimb ischemia induction, the rats were sacrificed by cervical dislocation after anesthesia with isoflurane, and the tissues of the ischemic and non-ischemic adductor muscles were harvested. We made 5-μm frozen sections of the tissues and incubated them with 2 mmol/L dihydroethidium (DHE) (Invitrogen), which is a probe to detect ROS production, for 30 min at 37°C in the dark. The DHE staining of the section was assessed in 5 randomly selected high-power fields (×200) under a fluorescence microscope (BX50, OLYMPUS). The DHE fluorescence intensity was quantified with image analysis computer software (Image-Pro Plus 6.2J, Media Cybernetics, Inc.).

### Limb Perfusion Measurement

At 14 days after the intramuscular injection of PBS with or without EPCs (5×10^5^ cells per rat) to the rat ischemic limb, we determined the blood flow of the rat ischemic and non-ischemic limbs with a laser Doppler blood flow imager (moorLDI-Mark 2, Moor Instruments Ltd.). Before scanning of the blood flow, rats were placed on a heating pad (FHP-300S, TGK) at 37°C to minimize variations in temperature. The mean of laser Doppler flux was analyzed on both limbs with software supplied by the manufacturer. To avoid variables due to ambient light and temperature, the calculated blood perfusion was expressed as the ischemic/non-ischemic hindlimb blood flow ratio [9], [19].

### Capillary Density Analysis

For histological assessment of neovascularization of the rat ischemic limb intramuscularly injected PBS with or without EPCs, we analyzed the capillary density in five-μm frozen sections of the ischemic adductor muscles at 14 days after the injection. Capillary endothelial cells were stained with CD31 monoclonal antibody (FITC-labeled, BD Biosciences). The numbers of CD31-positive capillaries (<20 μm), Dil-positive cells, and myofibers in 10 randomly selected high-power fields (×200) of the same traverse sections were counted with a fluorescence microscope (BX50, OLYMPUS). We divided the number of CD31-positive capillaries by the number of myofibers to calculate the CD31-positive capillary density [9], [19].

### EPCs Incorporation Analysis

For histological assessment of human-derived EPCs incorporation in endothelial cells of rat ischemic limbs with the intramuscular injection of the EPCs, we stained 5-μm frozen sections of the rat ischemic adductor muscles with CD31 monoclonal antibody (BD Pharmingen; labeled with Dylight549 IgG, Jackson Immunoresearch Laboratories) and proliferative cell nuclear antigen (PCNA) monoclonal antibody (abcam; labeled with HRP IgG, Molecular Probes and Alexa Fluor 488 Tyramide, Invitrogen). The CD31 antibody and PCNA antibody specifically react with human cells and rat cells, respectively. The numbers of PCNA-positive cells incorporated in CD31-positive endothelial cells (>10 μm) in 10 randomly selected high-power fields (×400) of the same traverse sections were counted with a fluorescence microscope (BZ-9000, Keyence).

### EPC Secretion Assay

EPCs secrete neovascularization-related cytokines [9], [19], [21]. In order to evaluate the secretion capacity of EPCs, we measured the concentrations of VEGF, b-FGF, SDF-1α, and IGF-1 in supplement-free culture medium with a high-sensitivity enzyme-linked immunosorbent assay kit (Amersham Pharmacia Biotech) [9], [19].

### Statistical Analysis

All values are expressed as means ± SE. In the western blot analysis, results of the expression of FOXO3a or FOXO4 in EPCs and H_2_O_2_-treated-EPCs were analyzed with the paired Student’s t-test. Results of the expressions of FOXO4 in healthy volunteer- and atherosclerotic patient-derived EPCs were analyzed with the Student’s t-test. Results of DHE tissue staining were also analyzed with the Student’s t-test. Other results were analyzed with one-way ANOVA with Tukey’s post-hoc test. P values <0.05 were considered statistically significant. All analyses were performed with SPSS version 16.0 (SPSS Inc.).

## Results

### Apoptosis of EPCs *in vitro*

**Figure 1:**
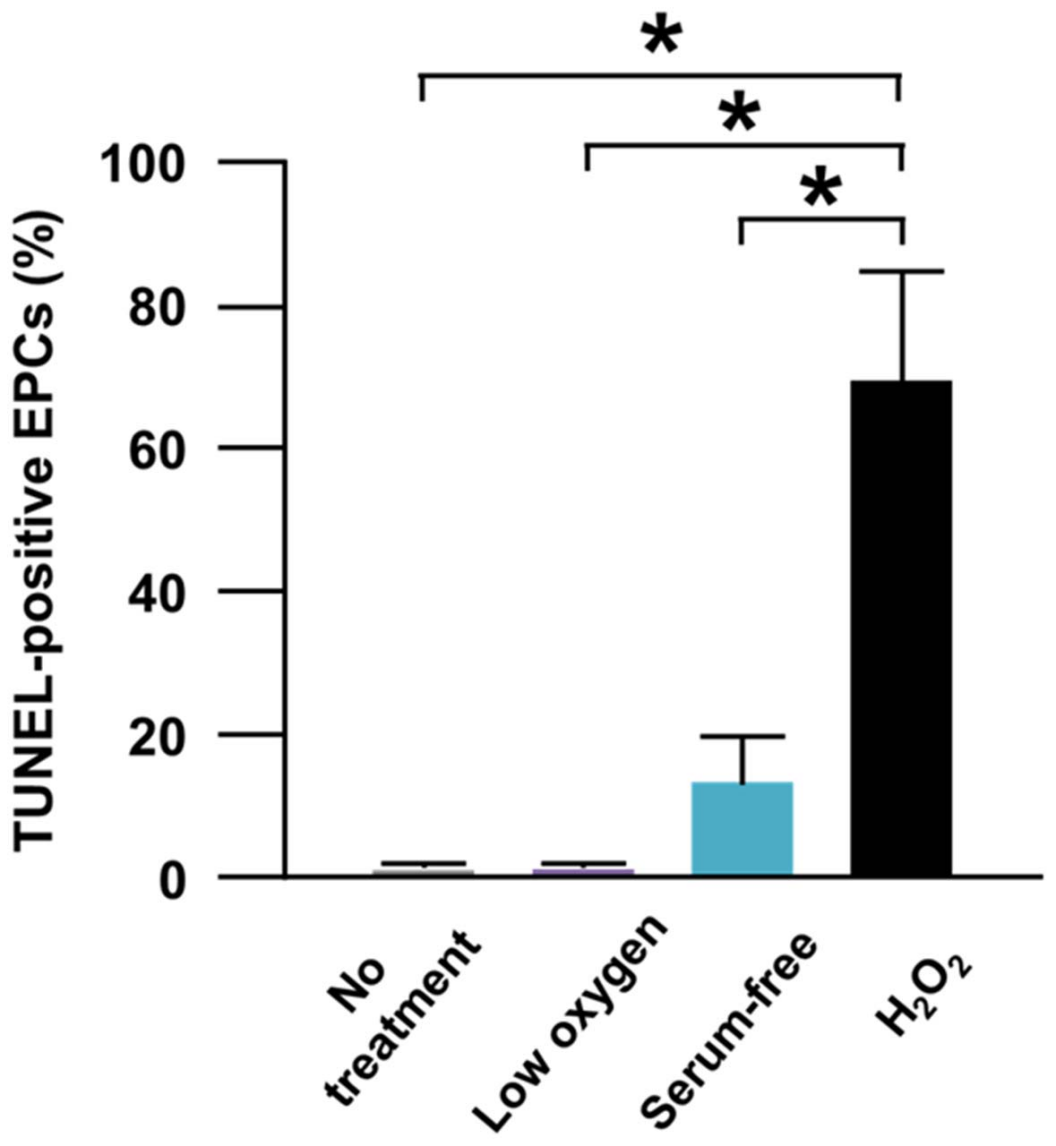
Apoptosis of EPCs subjected to cytotoxic condition *in vitro*. (A) Pooled data of the percentage of TUNEL-positive cells in the flow cytometric analysis for non-treated-EPCs, low oxygen-treated-EPCs, serum-free medium-treated-EPCs, and H_2_O_2_-treated-EPCs (*: p<0.001; n = 5, each).

In atherosclerotic patient-derived EPCs, the percentage of TUNEL-positive EPCs (i.e. apoptotic EPCs) was significantly greater for EPCs subjected to H_2_O_2_ (i.e. oxidative stress) than to serum-free medium (i.e. malnutrition stress) or to low oxygen (i.e. hypoxic stress). These results suggested high susceptibility of atherosclerotic patient-derived EPCs for oxidative stress-induced apoptosis (**Fig. 1**).

### FOXO Expressions in Apoptotic EPCs

**Figure 2:**
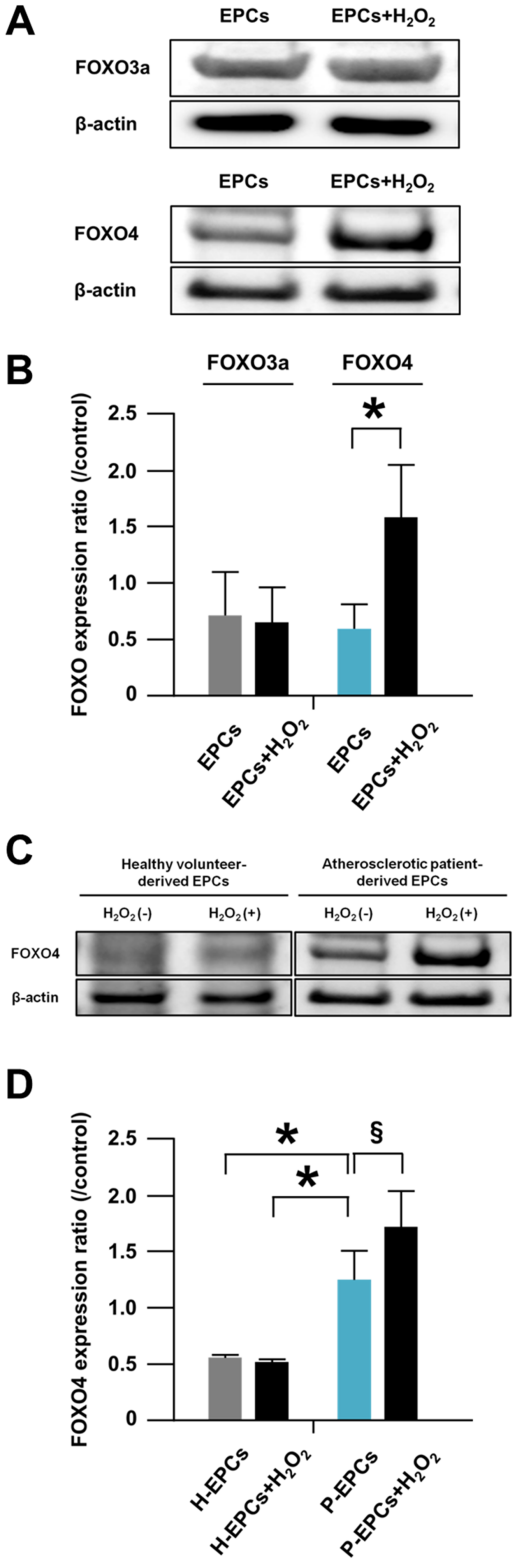
FOXO expressions in apoptotic EPCs. (A) Representative western blotting photos of expressions of FOXO3a and FOXO4 in EPCs and H_2_O_2_-treated-EPCs. EPCs were derived from atherosclerotic patients. (B) Pooled data of the FOXO3a/β-actin and FOXO4/β-actin expression ratios for the cells (*: p<0.05; n = 4, each). (C) A representative western blotting photo of expressions of FOXO4 in EPCs and H_2_O_2_-treated-EPCs. (D) Pooled data of the FOXO3a/β-actin and FOXO4/β-actin expression ratios of the cells. H-EPCs and P-EPCs indicate healthy volunteer-derived EPCs and atherosclerotic patient-derived EPCs, respectively (*: p<0.05; **^§^**: p<0.01; n = 5, each).

In atherosclerotic patient-derived EPCs, the expression of FOXO3a in H_2_O_2_-treated EPCs was comparable to that in non-treated EPCs (**Fig. 2A, B**). However, the expression of FOXO4 in H_2_O_2_-treated EPCs was significantly greater than that in non-treated EPCs (**Fig. 2A, B**). These results suggested that FOXO4 was more essential for oxidative stress-induced apoptosis of atherosclerotic patient-derived EPCs than FOXO3a.

Intriguingly, the expression of FOXO4 in EPCs under normal conditions was significantly greater for atherosclerotic patient-derived EPCs than for healthy volunteer-derived EPCs (**Fig. 2C, D**). The baseline clinical characteristics of healthy volunteer- and atherosclerotic patient-derived EPCs are shown in **Table 1**. Moreover, the expression of FOXO4 in EPCs subjected to H_2_O_2_ was augmented in atherosclerotic patient-derived EPCs only (**Fig. 2C, D**). These results suggested again that FOXO4 was essential for oxidative stress-induced apoptosis of atherosclerotic patient-derived EPCs.

**Table 1:** Baseline clinical characteristics of healthy volunteer- and atherosclerotic patient-derived EPCs.

	Healthy volunteers	Atherosclerotic patients	
	(n = 10)	(n = 10)	
Age (yrs)	34.4±1.4	74.2±2.7	P<0.001
Gender			
Male	8	8	N.S
Female	2	2	N.S
Hypertension	0	9	P<0.001
Dyslipidemia	0	8	P<0.005
Diabetes mellitus	0	4	P<0.05
Smoking	2	7	P<0.05
Medication			
RASi	0	5	P<0.05
Statin	0	8	P<0.001

RASi = renin-angiotensin system inhibitor. Data are presented as the mean ± SEM.

Taken together, we adopted FOXO4 to generate FOXO-knockdown EPCs gained resistance to oxidative stress-induced apoptosis and used atherosclerotic patient-derived cells for the generation.

### Generation of FOXO4^KD^-EPCs

**Figure 3:**
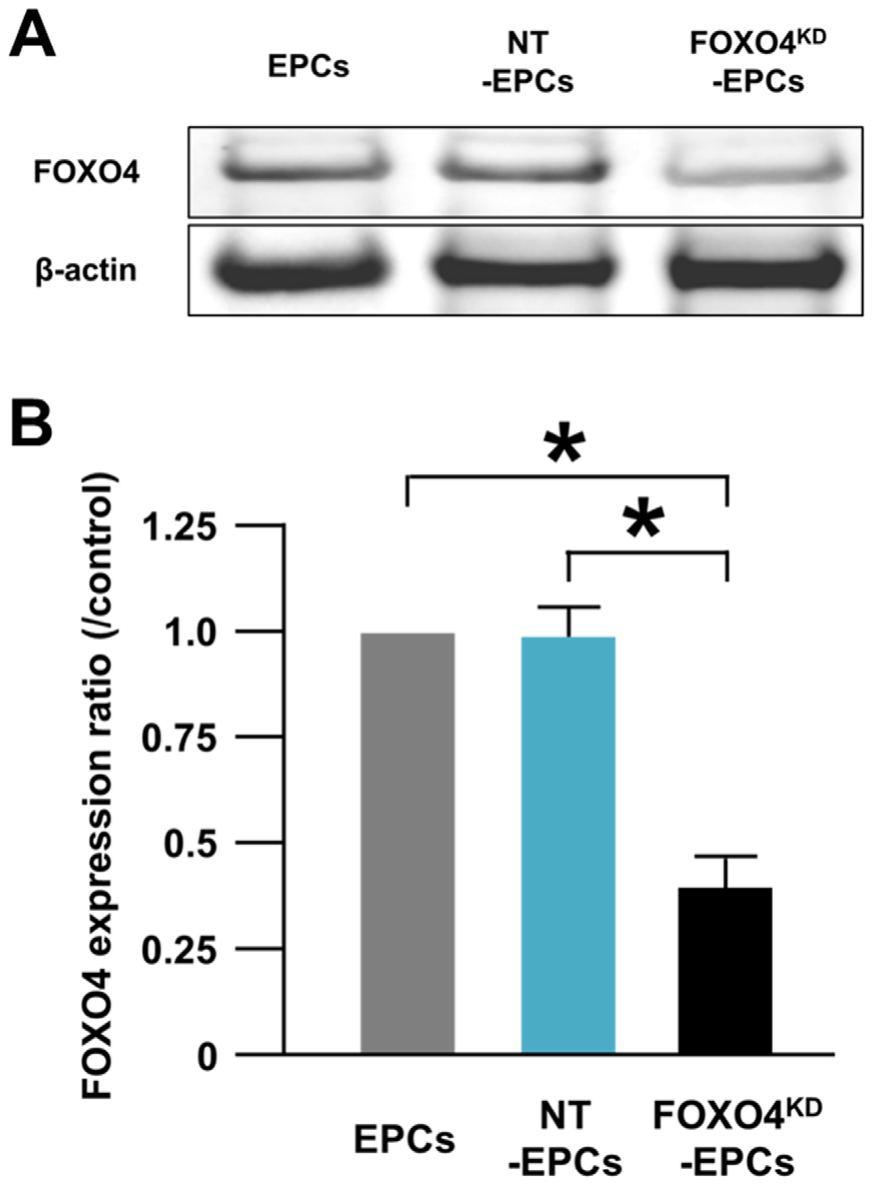
Generation of FOXO4^KD^-EPCs. (A) A representative western blotting photo of expressions of FOXO4 in EPCs, NT-EPCs, and FOXO4^KD^-EPCs. (B) Pooled data of the FOXO4/β-actin expression ratios of the cells (*: p<0.001; n = 5, each).

FOXO4 expression in negative control siRNA transfected EPCs (NT-EPCs) was comparable to that in non-transfected EPCs (**Fig. 3A**). FOXO4 expression in FOXO4 siRNA transfected EPCs was significantly lower by 39.3±7.2% than that in non-transfected EPCs, indicating successful generation of FOXO4^KD^-EPCs (**Fig. 3B**).

### Phenotype of EPCs

**Figure 4:**
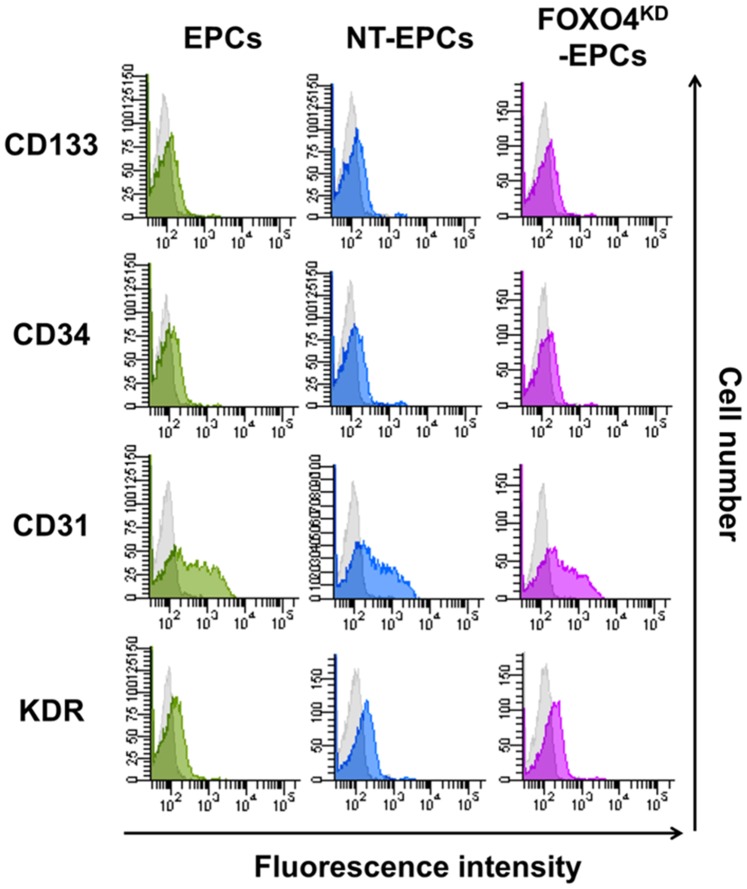
Phenotype of FOXO4^KD^-EPCs. Representative histograms of the flow cytometric analysis for EPC surface antigens between EPCs (green histograms), NT-EPCs (blue histograms), or FOXO4^KD^-EPCs (pink histograms). The gray histograms indicate isotype-matched IgG. Comparable results were shown in five experiments.

In the flow cytometric analysis, the expressions of CD133, CD34, CD31, and KDR, which were reported as surface antigens of EPCs [3], [9], [19], were comparable among EPCs, NT-EPCs, and FOXO4^KD^-EPCs (**Fig. 4**). These results indicated that siRNA transfection did not alter the phenotype of EPCs.

### Anti-apoptosis Capacity of FOXO4^KD^-EPCs *in vitro*

**Figure 5:**
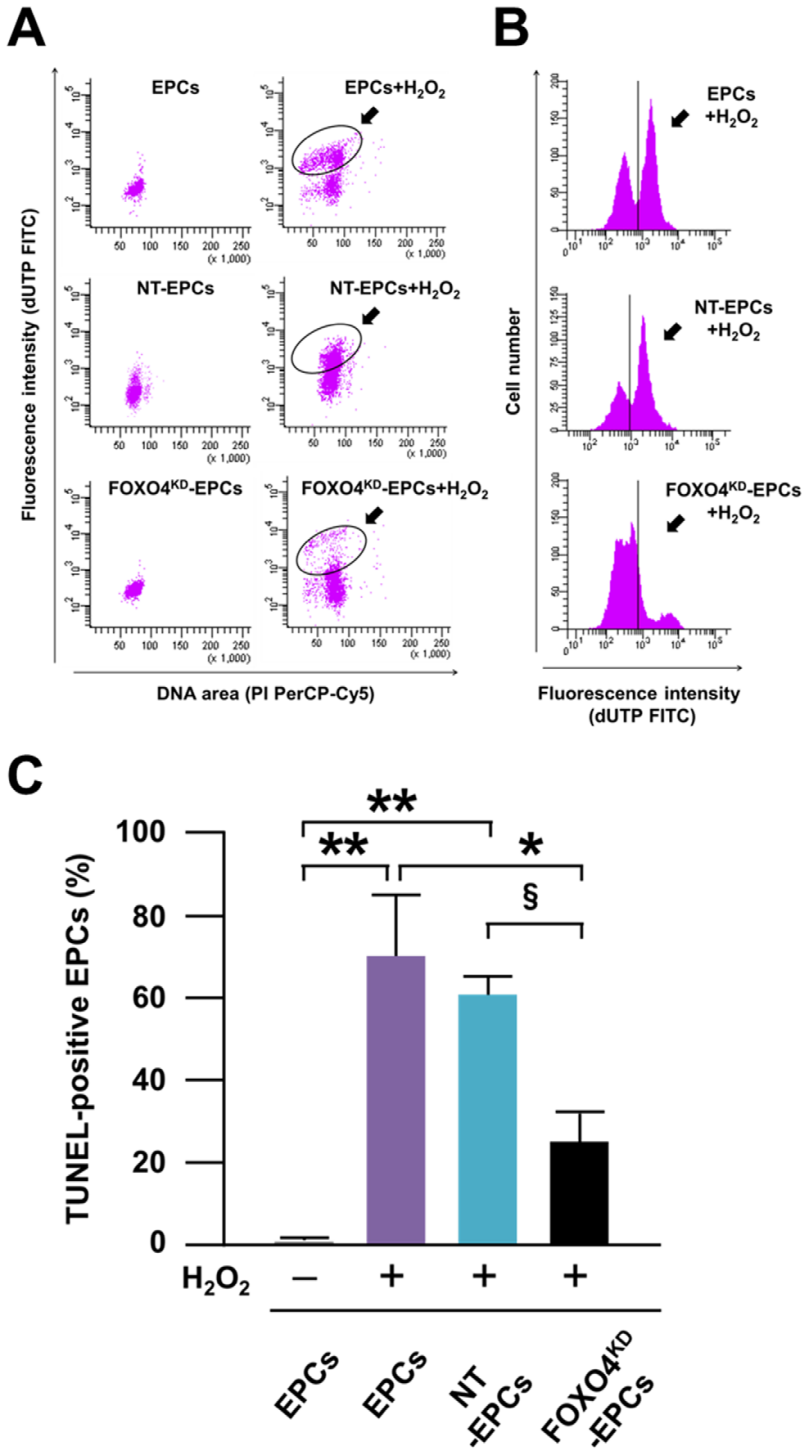
Anti-apoptosis capacity of FOXO4^KD^-EPCs *in vitro*. Representative dot plots (A) and histograms (B) of the flow cytometric analysis for EPCs and H_2_O_2_-treated-EPCs followed by TUNEL staining. The dot plots indicate EPCs positive for deoxyuridiine triphosphate (dUTP) and/or propidium iodide (PI). The dUTP-positive (i.e. TUNEL-positive) cells and PI-positive cells mean apoptotic and dead cells, respectively. The two black circles are located on the same coordinate axis of flow cytometry. The black vertical line on the histogram corresponds to the peak channel of fluorescence intensity of the isotype-matched IgG control. (C) Pooled data of the percentage of TUNEL-positive cells in the flow cytometric analysis for EPCs (**: p<0.001; *: p<0.01; ^§^: p<0.05; n = 5–6, each).

**Figure 6:**
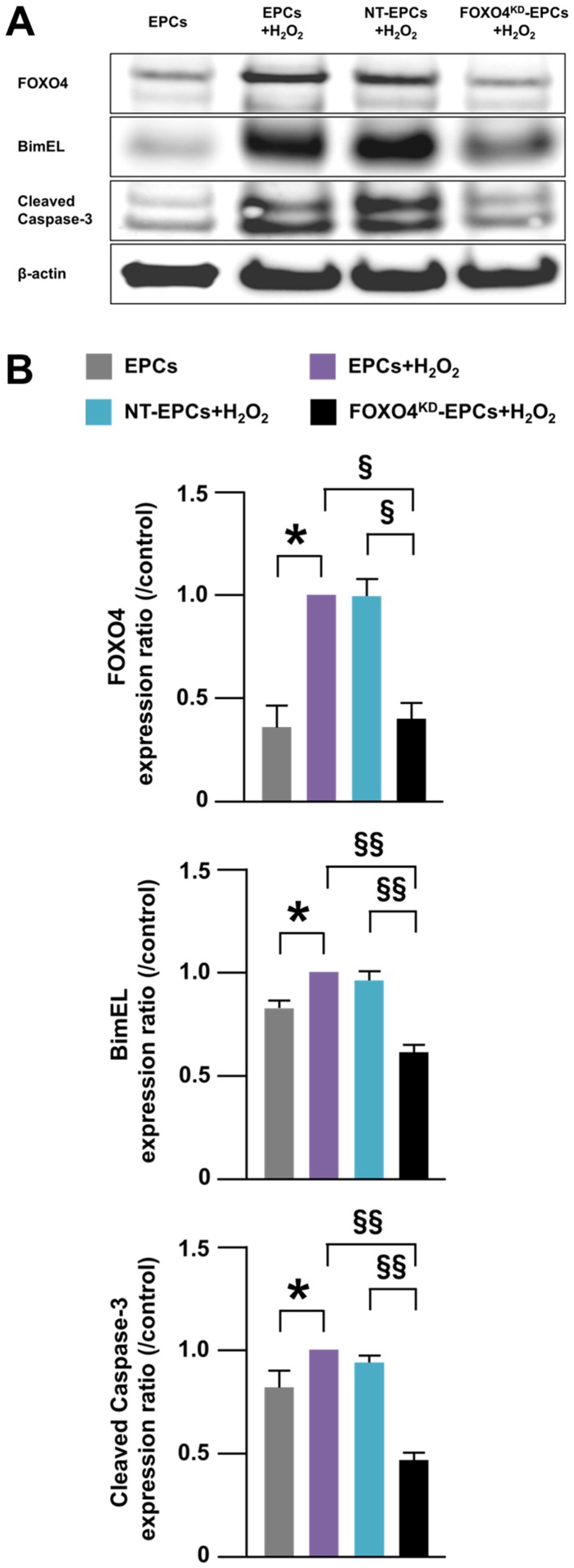
Expressions of pro-apoptotic proteins of FOXO4^KD^-EPCs. (A) A representative western blotting photo of expressions of FOXO4, BimEL, and cleaved caspase-3 in EPCs. (B) Pooled data of the FOXO4/β-actin, BimEL/β-actin, and cleaved caspase-3/β-actin expression ratios for EPCs. The expression ratios in non-transfected and H_2_O_2_-treated EPCs (purple bars) were control. (*: p<0.05; ^§^: p<0.0005;^ §§^: p<0.0001; n = 5–7, each).

After treatment of EPCs with H_2_O_2_, the number of TUNEL-positive EPCs was significantly smaller for FOXO4^KD^-EPCs than for EPCs and NT-EPCs (**Fig. 5A–C**). The expressions of BimEL and cleaved caspase-3, which were reported as pro-apoptotic proteins of EPCs [18], [22], were significantly decreased as well as the expression of FOXO4 in FOXO4^KD^-EPCs treated with H_2_O_2_ (**Fig. 6A,B**). These results suggested that anti-apoptosis capacity for oxidative stress was augmented in FOXO4^KD^-EPCs.

### ROS Production in Athymic Nude Rat Ischemic Limbs

**Figure 7:**
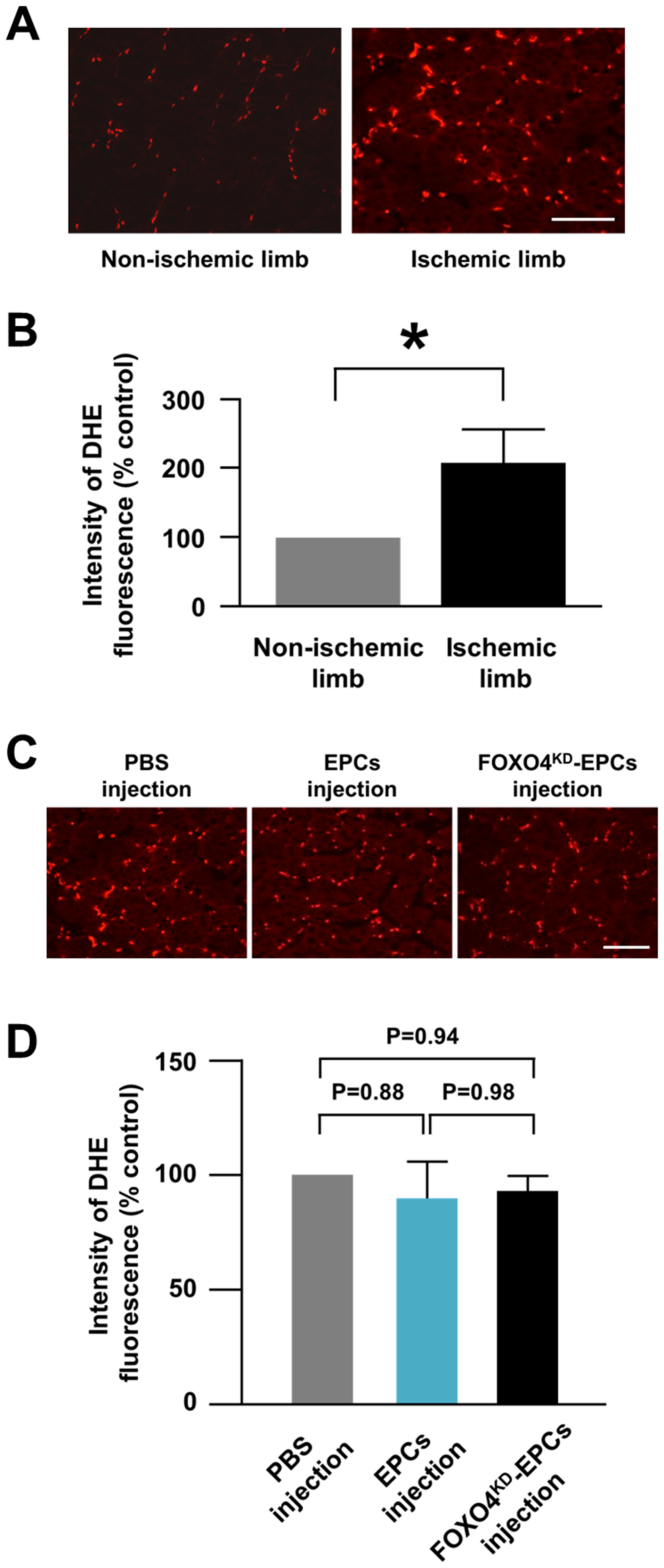
ROS production in athymic nude rat ischemic limbs. (A) Representative fluorescence microscopic images of DHE-stained tissues of the non-ischemic and ischemic limbs of athymic nude rats. DHE was stained red. Scale bar: 100 μm. (B) Pooled data of DHE fluorescence intensity of the rat non-ischemic and ischemic limbs (*: p<0.05; n = 12, each). (C) Representative fluorescence microscopic images of DHE-stained tissues of the ischemic limbs 24 h after intramuscular injection of PBS, EPCs, or FOXO4^KD^-EPCs. Scale bar: 100 μm. (D) Pooled data of DHE fluorescence intensity of the ischemic limbs 24 h after intramuscular injection of PBS, EPCs, or FOXO4^KD^-EPCs (n = 7, each).

In the rat ischemic and contralateral non-ischemic limb tissues stained with DHE, the fluorescence intensity of DHE was significantly greater for the ischemic tissues than for the non-ischemic tissues, indicating that more ROS was produced in the ischemic limb than in the non-ischemic limb (**Fig. 7A, B**). In addition, the DHE expression in the ischemic limb injected EPCs or FOXO4^KD^-EPCs was comparable to that in the ischemic limb injected PBS (**Fig. 7C, D**). These results suggested that intramuscularly injected EPCs or FOXO4^KD^-EPCs did not reduce ROS in the ischemic limb tissues.

### Anti-apoptosis Capacity of FOXO4^KD^-EPCs *in vivo*

**Figure 8:**
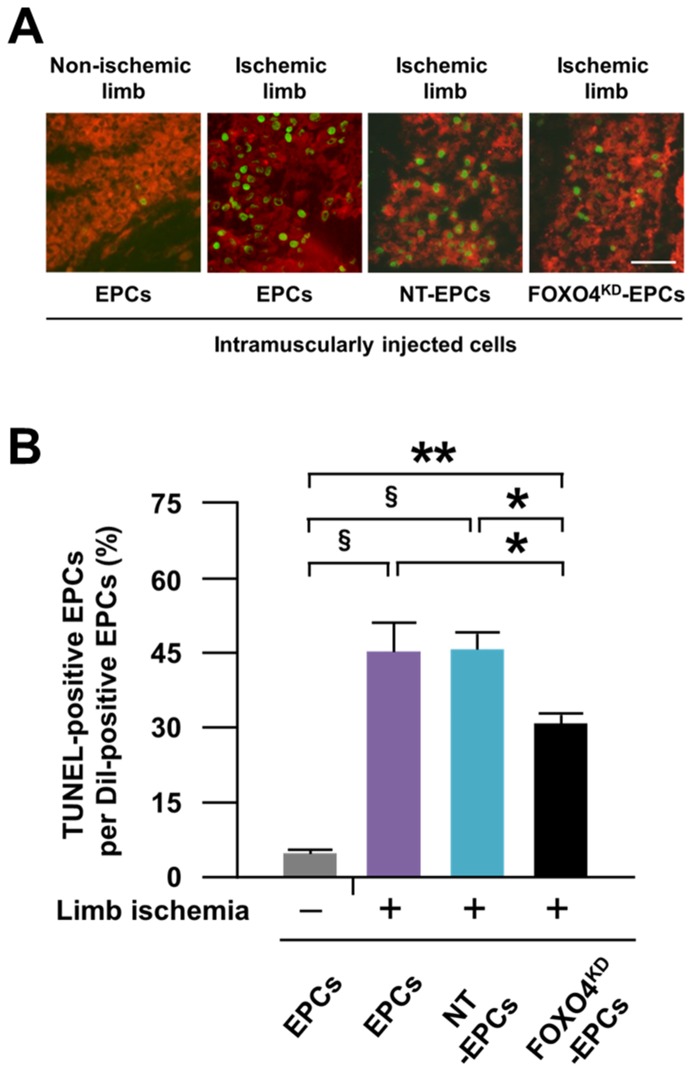
Anti-apoptosis capacity of FOXO4^KD^-EPCs *in vivo*. (A) Representative fluorescence microscopic photos of Dil-labeled cells 24 h after intramuscular injection to the rat non-ischemic or ischemic limb. The red cells and green cells indicate Dil-labeled EPCs and TUNEL-positive cells, respectively. (B) Pooled data of the apoptosis ratio for the injected cells (*: p<0.05; **: p<0.0005;^ §^: p<0.0001; n = 5–6, each).

At 24 h after intramuscular injection of EPCs to the rat ischemic and contralateral non-ischemic limbs, significantly more EPCs were positive for TUNEL in the ischemic limb than in the non-ischemic limb (**Fig. 8A, B**), indicating more apoptosis of the injected EPCs in the ischemic limb than in the non-ischemic limb. However, the number of apoptotic EPCs was significantly smaller for FOXO4^KD^-EPCs but not NT-EPCs in the ischemic limb (**Fig. 8A, B**), suggesting the augmented anti-apoptosis capacity of FOXO4^KD^-EPCs in the ischemic limb.

### Neovascularization Capacity of FOXO4^KD^-EPCs *in vivo*

**Figure 9:**
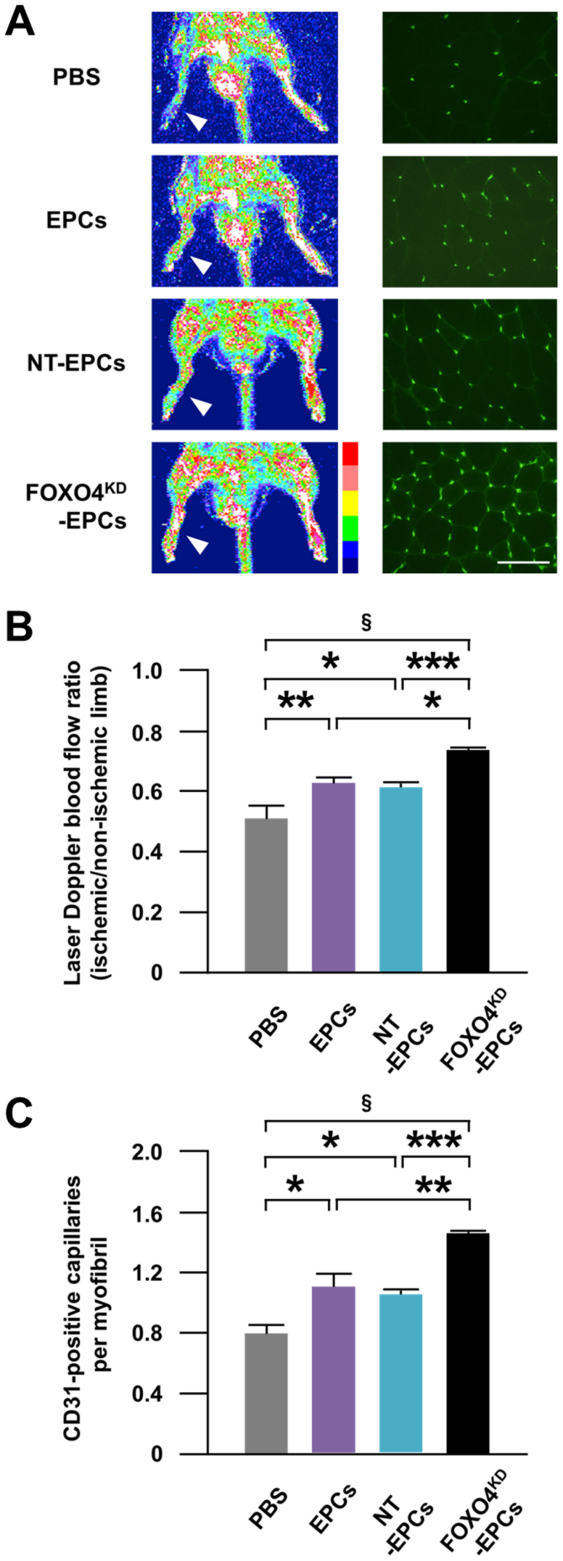
Neovascularization capacity of FOXO4^KD^-EPCs *in vivo*. (A) Representative laser Doppler blood flow images of both limbs of athymic nude rats (left panels) and fluorescence microscopic photos of CD31-positive endothelial capillaries in the ischemic limb (right panels) 14 days after intramuscular injection of PBS, EPCs, NT-EPCs, or FOXO4^KD^-EPCs to the right ischemic limb. The hindlimbs enclosed by white squares indicate the right ischemic limbs. Red to white color and dark blue color on the image indicate high and low perfusion signals, respectively. CD31-positive endothelial capillaries were stained green. Scale bar: 100 μm. Pooled data of the ischemic/non-ischemic hindlimb blood flow ratio (B) and the capillary density (C) 14 days after the injection (*: p<0.05; **: p<0.01; ***: p<0.005;^ §^: p<0.0005; n = 5–6, each).

At 14 days after intramuscular injection of PBS or EPCs to the rat ischemic limb, the blood flow ratio and the CD31-positive capillary density of the ischemic limb were significantly greater for the injection of EPCs than for the injection of PBS (**Fig. 9A–C**), which were further increased by the injection of FOXO4^KD^-EPCs, but not NT-EPCs (**Fig. 9A–C**).

### Incorporation of EPCs in Endothelial Cells *in vivo*

**Figure 10:**
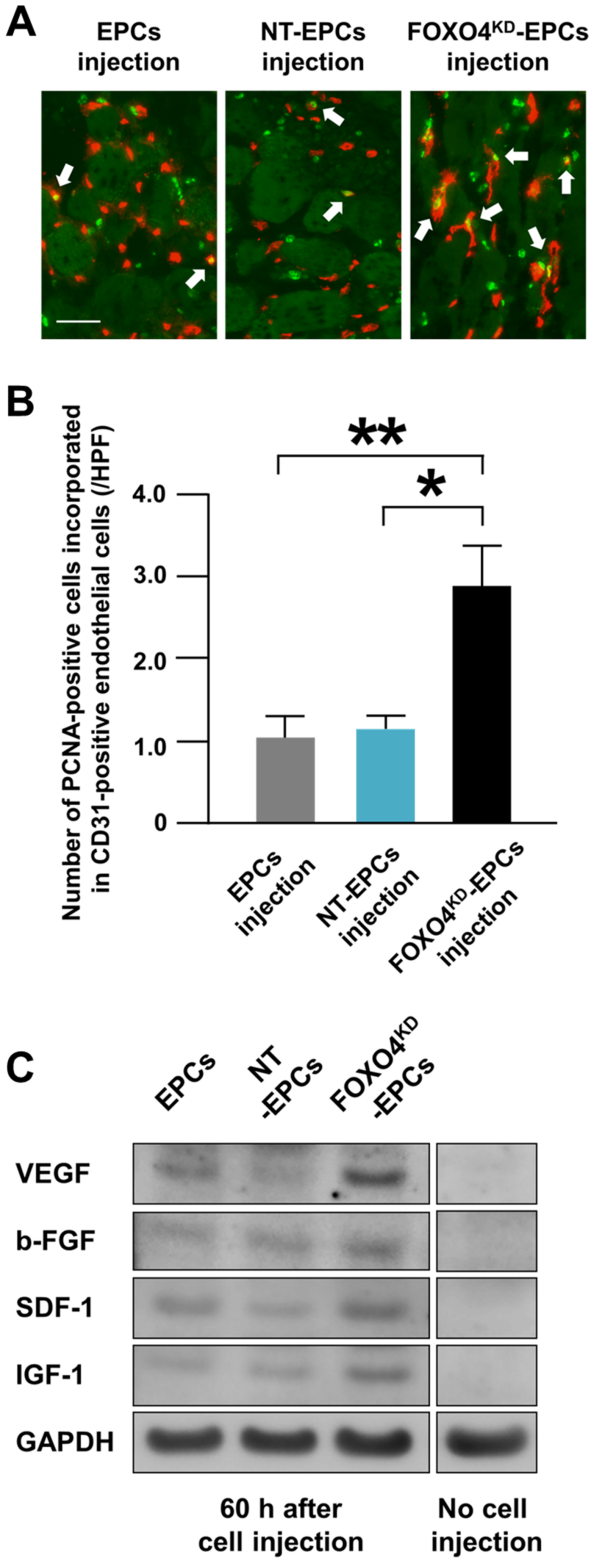
Kinetics of EPCs for neovascularization *in vivo*. (A) Representative fluorescence microscopic images of EPCs incorporated in endothelial cells of the ischemic limbs of athymic nude rats 60 h after intramuscular injection of EPCs, NT-EPCs, or FOXO4^KD^-EPCs. Rat specific CD31-positive endothelial cells were stained red. Human specific PCNA-positive cells, which were defined as human-derived EPCs, were stained green. Scale bar: 50 μm. (B) Pooled data of the number of PCNA-positive EPCs incorporated in endothelial cells of the rat ischemic limbs 60 h after the injection of EPCs, NT-EPCs, or FOXO4^KD^-EPCs (*: p<0.01; **: p<0.005; n = 5, each). (C) A representative western blotting photo of expressions of human specific VEGF, b-FGF, SDF-1, and IGF-1 in the rat ischemic limb 60 h after intramuscular injection of EPCs, NT-EPCs, or FOXO4^KD^-EPCs (n = 4, each).

At 60 h after intramuscular injection of human-derived EPCs in the rat ischemic limb, human-specific proliferative cell nuclear antigens (PCNA)-positive cells, which were defined as injected EPCs in proliferative phase, were incorporated in rat CD31-positive endothelial cells (**Fig. 10A**). Moreover, the number of PCNA-positive cells incorporated in CD31-positive endothelial cells was significantly greater in the injection of FOXO4^KD^-EPCs than in the injections of EPCs and NT-EPCs (**Fig. 10B**). This result suggested that more FOXO4^KD^-EPCs survived in the ischemic limb tissue and were incorporated in endothelial cells of the tissue for augmenting neovascularization of the limb.

### Secretion Capacity of FOXO4^KD^-EPCs

The concentrations of VEGF, b-FGF, IGF-1, and SDF-1α secreted from EPCs in the culture medium were comparable among EPCs, NT-EPCs, and FOXO4^KD^-EPCs (**Table 2**), suggesting that there were no effect of FOX4-knockdown on the secretion capacity of EPCs.

**Table 2:** Concentrations of EPC-secreted angiogenic proteins in the culture medium.

		EPCs	NT-EPCs	FOXO4^KD^-EPCs	
		(n = 6)	(n = 6)	(n = 6)	
VEGF	(×10 pg/ml)	13.8±2.5	11.4±0.5	12.3±0.9	N.S
b-FGF	(×10 pg/ml)	1.4±0.4	2.2±0.3	2.1±0.1	N.S
IGF-1	(×10 pg/ml)	27.1±5.6	17.4±1.2	17.9±1.6	N.S
SDF-1α	(×10 pg/ml)	82.5±17.2	95.9±13.7	87.4±20.7	N.S

VEGF: vascular endothelial growth factor; b-FGF: basic-fibroblast growth factor;

IGF-1: insulin-like growth factor-1; SDF-1α: stromal cell derived factor-1α.

### Angiogenic Cytokines in Athymic Nude Rat Ischemic Limbs

We examined EPC-secreted angiogenic cytokines such as VEGF, b-FGF, IGF-1, and SDF-1 in the rat ischemic limb using western blot analysis. At 60 h after the EPCs injection, the expressions of human specific VEGF, b-FGF, SDF-1, and IGF-1 were greater in the injection of FOXO4^KD^-EPCs than in the injections of EPCs and NT-EPCs (**Fig. 10C**). These results suggested that more FOXO4^KD^-EPCs survived in the ischemic limb tissue and secreted more angiogenic cytokines in the tissue for augmenting neovascularization of the limb.

## Discussion

### Oxidative Stress Enhanced Apoptosis of EPCs *in vivo*

**Figure 11:**
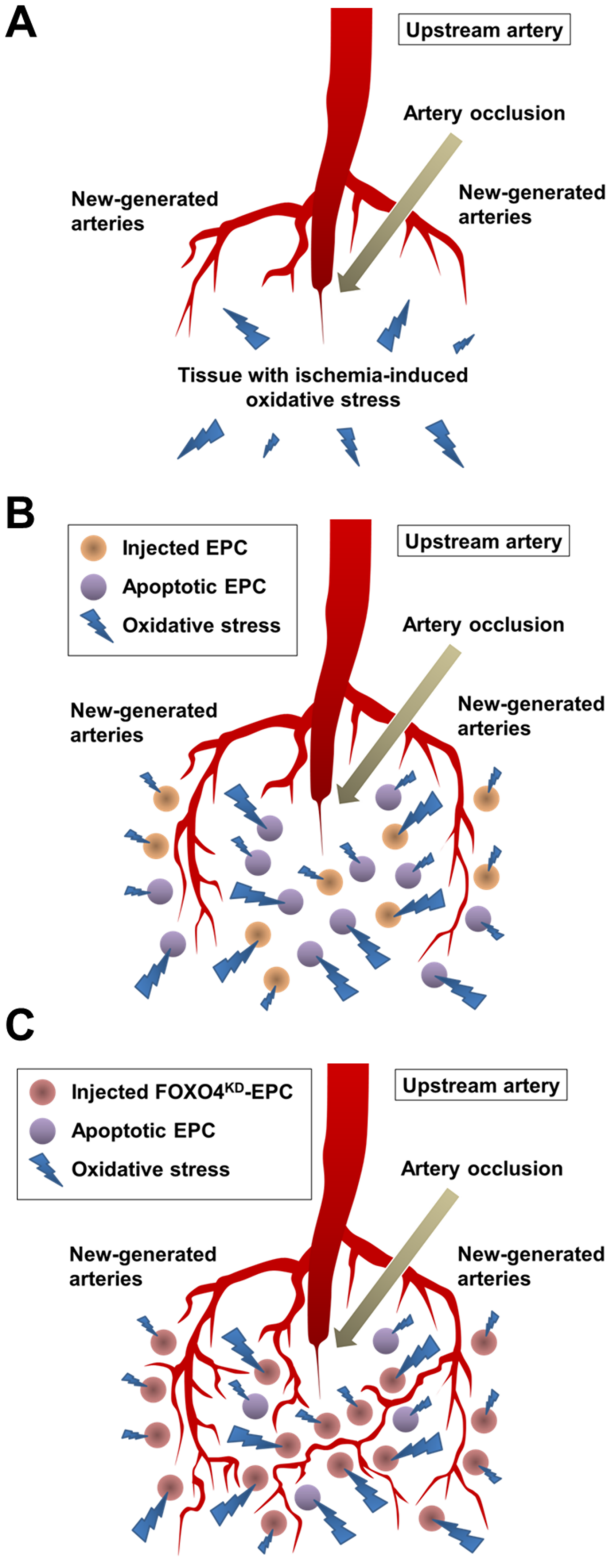
Imaginable schemas of the neovascularization in rat ischemic limb tissues with ischemia-induced oxidative stress. (A) An imaginable schema the neovascularization in the tissues without EPCs injection, (B) with EPCs injection, and (C) with FOXO4^KD^-EPCs injection.

Arterial occlusion of the limb causes tissue ischemia, subsequently resulting in depletion of the energy, collapse of the ion equilibrium, and induction of oxidative stress for the limb [11]–[13]. EPCs have been injected to such ischemic limb for therapeutic angiogenesis so far. In this study, oxidative stress was higher cytotoxic factor for EPCs than malnutrition and hypoxic stress *in vitro*. This result may suggest that a great number of EPCs that are injected to the ischemic limb lose the viability due to ischemia-induced oxidative stress in the limb (**Fig. 11A, B**). Oxidative stress, which is caused by an imbalance between the production and accumulation of ROS and the body’s ability to manage them with various antioxidants, was reported to be increase in ischemic skeletal muscles of human and rodent limbs [11]–[13]. In this study, the expression of DHE, which is a probe to detect ROS production, was greater in the ischemic limb of athymic nude rat than in the contralateral non-ischemic limb of the rat, indicating that oxidative stress was increased in the rat ischemic limb. Moreover, at 24 h after the intramuscular injection of EPCs to the rat limb with or without ischemia, the number of apoptotic EPCs was greater in the ischemic limb than in the non-ischemic limb. These results suggested that intramuscularly injected EPCs to the ischemic limb resulted in apoptosis partly due to oxidative stress of the limb and a great number of viable EPCs with neovascularization capacity were lost from the limb.

### FOXO4-knockdown Suppressed Oxidative Stress-induced Apoptosis of EPCs *in vitro*

It has been reported that oxidative stress activated FOXO and the activation promoted apoptosis [16], [17]. In this study, the expression of FOXO4, but not FOXO3a, was augmented in H_2_O_2_-treated EPCs, which may suggest that oxidative stress induces FOXO4-mediated apoptosis of EPCs. In addition, FOXO4^KD^-EPCs prevented oxidative stress-induced apoptosis *in vitro* and the expressions of pro-apoptotic proteins BimEL and cleaved caspase-3 were decreased in FOXO4^KD^-EPCs in response to oxidative stress. Thus, we succeeded *ex vivo* in generating FOXO4^KD^-EPCs with augmented anti-apoptosis capacity for oxidative stress.

### FOXO4^KD^-EPCs Contributed to Augment Neovascularization *in vivo*

In order to assess whether the *ex vivo* anti-apoptosis capacity of FOXO4^KD^-EPCs could work *in vivo*, we intramuscularly injected EPCs, NT-EPCs, or FOXO4^KD^-EPCs into the rat ischemic limb and assessed neovascularization of the limb. At 24 h after the cell injection, the number of apoptotic FOXO4^KD^-EPCs was significantly smaller than that of apoptotic EPCs or NT-EPCs in the limb. The the injection of EPCs or NT-EPCs increased the blood flow and the capillary density of the rat ischemic limb more than the injection of PBS at 14 days, which were further increased by the injection of FOXO4^KD^-EPCs. Moreover, injected EPCs in proliferative phase were incorporated in endothelial cells of the limb tissue and the number of the EPCs was significantly greater in the injection of FOXO4^KD^-EPCs than in the injections of EPCs and NT-EPCs. These results suggested that the *ex vivo* anti-apoptosis capacity of FOXO4^KD^-EPCs could work in the rat ischemic limb to decrease the loss of intramuscularly injected EPCs and to let the surviving many EPCs fully bring out the neovascularization capacity in the limb (**Fig. 11B, C**). Urbich et al. reported that EPCs secreted angiogenic proteins such as VEGF, SDF-1, and IGF-1 and thereby enhanced migration of mature endothelial cells for neovascularization of the ischemic tissue [21]. In this study, FOXO4-knockdown did not affect the secretion of such angiogenic proteins. Given the augmented anti-apoptosis capacity of FOXO4^KD^-EPCs in the ischemic limb, the amount of angiogenic proteins secreted from intramuscularly injected EPCs was greater for FOXO4^KD^-EPC-injected limb than for non-treated EPC-injected limb. This may be one of the mechanisms of augmented neovascularization by the injection of FOXO4^KD^-EPCs. Indeed, the expression of VEGF, b-FGF, SDF-1, and IGF-1 secreted from EPCs in the rat ischemic limb tissue were greater in the injection of FOXO4^KD^-EPCs than in the injections of EPCs and NT-EPCs.

Urao et al. have recently reported in endothelial specific catalase transgenic mice with hindlimb ischemia that endothelial cell-derived H_2_O_2_, which is a kind of ROS, plays a critical role in neovascularization of the ischemic limb [23]. Although we assume in this study that ischemia-induced oxidative stress plays an undesirable role in EPC-mediated neovascularization of the ischemic limb, there may be the possibility that intramuscularly injected EPCs reduced endothelial cell-derived H_2_O_2_ in the ischemic limb and thereby impaired endothelial cell-regulated neovascularization. However, this possibility was thought to be unlikely because the expression of DHE in EPC- or FOXO4^KD^-EPC-injected ischemic limb tissue was comparable to that in PBS-injected ischemic limb tissue. Thus, FOXO4^KD^-EPCs injection to the ischemic limb did not affect neovascularization regulated by the endothelial cell-derived ROS but contributed to augment EPC-mediated neovascularization by the decrease of oxidative stress-induced apoptosis of EPCs.

### Study Limitations

There were several limitations in this study. First, we used EPCs, but not MNCs, in this study. Further, EPCs were generated from PB-MNCs, but not from BM-MNCs. FOXO4-knockdown in BM-MNCs should be examined in our future study. Second, we did not directly examine the activation of FOXO4 (e.g. the expression of phosphorylated FOXO4) in apoptotic EPCs but indirectly assessed the activation by expressions of BimEL and cleaved caspasse-3. Third, we assessed the *in vivo* neovascularization capacity of FOXO4^KD^-EPCs in the ischemic limbs of rats without atherosclerotic risk factors. It has been reported that hindlimb ischemia induction in rodents with atherosclerotic risk factors further increased oxidative stress in the plasma and ischemic limb tissue [24]–[26]. Accordingly, in the ischemic limbs of rodents with atherosclerotic risk factors we may not be able to obtain the same neovascularization by FOXO4^KD^-EPCs as we did in this study. Forth, we did not perform this study using healthy volunteer-derived FOXO4^KD^-EPCs *in vivo*. Although the expression of FOXO4 in EPCs subjected to H_2_O_2_ *in vitro* was augmented in atherosclerotic patient-derived EPCs only, future studies are required to examine if the *in vivo* neovascularization capacity of healthy volunteer-derived EPCs was augmented by FOXO4-knockdown of the cells as observed in atherosclerotic patient-derived EPCs. However, most critical limb ischemia patients undergoing therapeutic angiogenesis have several atherosclerotic risk factors and the patient-derived cells are actually used in the therapy, suggesting an appropriate use of atherosclerotic patient-derived cells for this study. Fifth, we were not able to examine the anti-apoptosis capacity of FOXO4^KD^-EPCs *in vivo* in several time points after intramuscular injection to the rat ischemic limb, because we were not able to find any TUNEL-positive EPCs in the limb 72 h after the injection of Dil-labeled EPCs (data not shown), suggesting that apoptotic EPCs in the limb might be phagocytized by cells such as macrophages within 72 h of the injection. Thus, it may be difficult to study the anti-apoptosis capacity of FOXO4^KD^-EPCs after 24 h of the injection to ischemic tissues. Sixth, we have performed this study using hydrogen peroxide, but not other stimulators. Future studies are required to examine if other stimulators and transcriptional regulators, which were induced by hypoxic condition in the rat ischemic limb, related to apoptosis of EPCs. We do not consider that hydrogen peroxide is the main source to induce apoptosis of EPCs intramuscularly injected in the ischemic limb.

## Conclusion

FOXO4^KD^-EPCs gained resistance to oxidative stress-induced apoptosis in the ischemic limb, resulting in decrease of the loss of workable EPCs for neovascularization of the limb. Intramuscular injection of FOXO4^KD^-EPCs may be a promising therapeutic strategy for patients with critical limb ischemia.
