# Correction: FOXO4-Knockdown Suppresses Oxidative Stress-Induced Apoptosis of Early Pro-Angiogenic Cells and Augments Their Neovascularization Capacities in Ischemic Limbs

**DOI:** 10.1371/journal.pone.0127245

**Published:** 2015-04-27

**Authors:** Takaharu Nakayoshi, Ken-ichiro Sasaki, Hidemi Kajimoto, Hiroshi Koiwaya, Masanori Ohtsuka, Takafumi Ueno, Hidetoshi Chibana, Naoki Itaya, Masahiro Sasaki, Shinji Yokoyama, Yoshihiro Fukumoto, Tsutomu Imaizumi

Following the publication of the article, the following errors were identified in two of the figures:

- The FOXO4 panel Fig 2A was duplicated as the right panel in Fig 2C.
- The right panel of Fig 7A incorrectly displays the flip vertical image of the left panel in Fig 7C.

The authors apologize for these errors.

Additional experiments have been carried out to assess FOXO4 expression in atherosclerotic patient-derived early pro-angiogenic cells from additional patients with or without H2O2 pretreatment (n = 13), the results from these experiments support the conclusions reported in the article.

The authors are providing a new Fig 2 reporting the results of the replication experiments as well as a corrected Fig 7.

**Fig 2:**
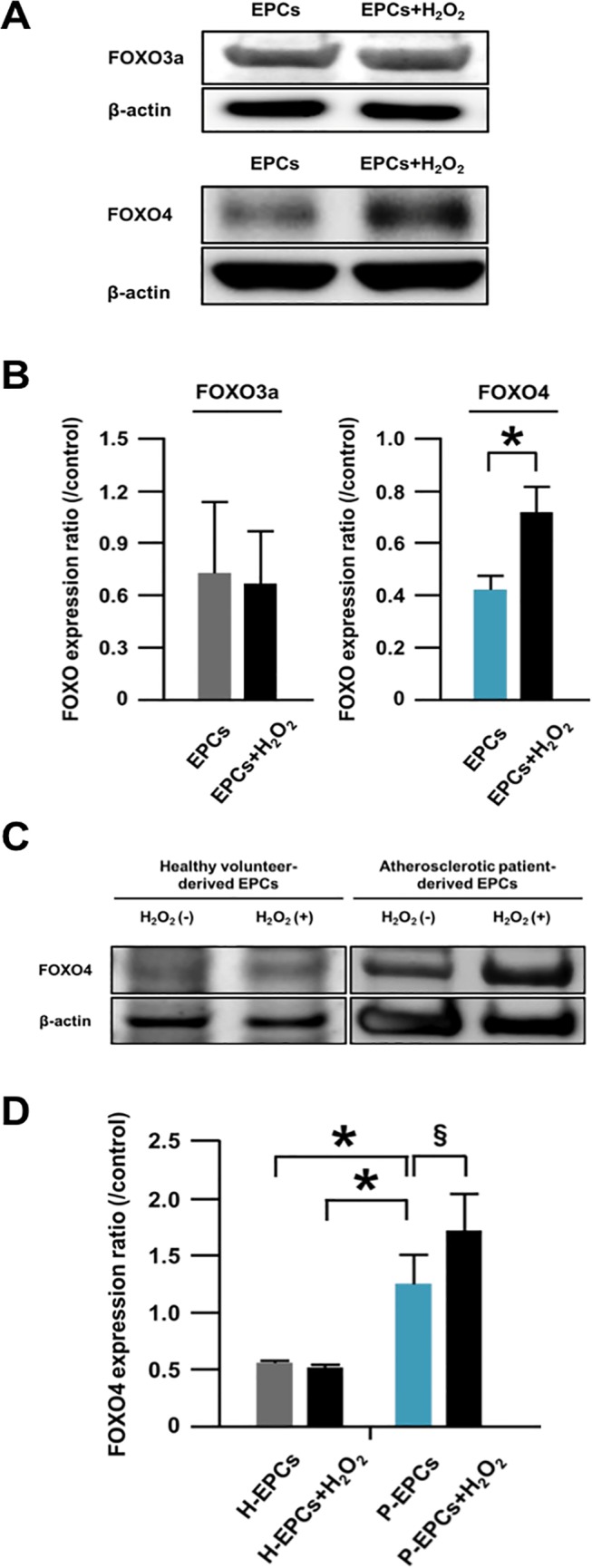
FOXO expressions in apoptotic EPCs. (A) Representative western blotting photos of expressions of FOXO3a and FOXO4 in EPCs and H_2_O_2_-treated-EPCs. EPCs were derived from atherosclerotic patients. (B) Pooled data of the FOXO3a/β-actin and FOXO4/β-actin expression ratios for the cells (*: p<0.005; n = 4–13, each). (C) A representative western blotting photo of expressions of FOXO4 in EPCs and H_2_O_2_-treated-EPCs. (D) Pooled data of the FOXO3a/β-actin and FOXO4/β-actin expression ratios of the cells. H-EPCs and P-EPCs indicate healthy volunteer-derived EPCs and atherosclerotic patient-derived EPCs of a different group from figures 2A and B, respectively (*: p<0.05; §: p<0.01; n = 5, each).

**Fig 7:**
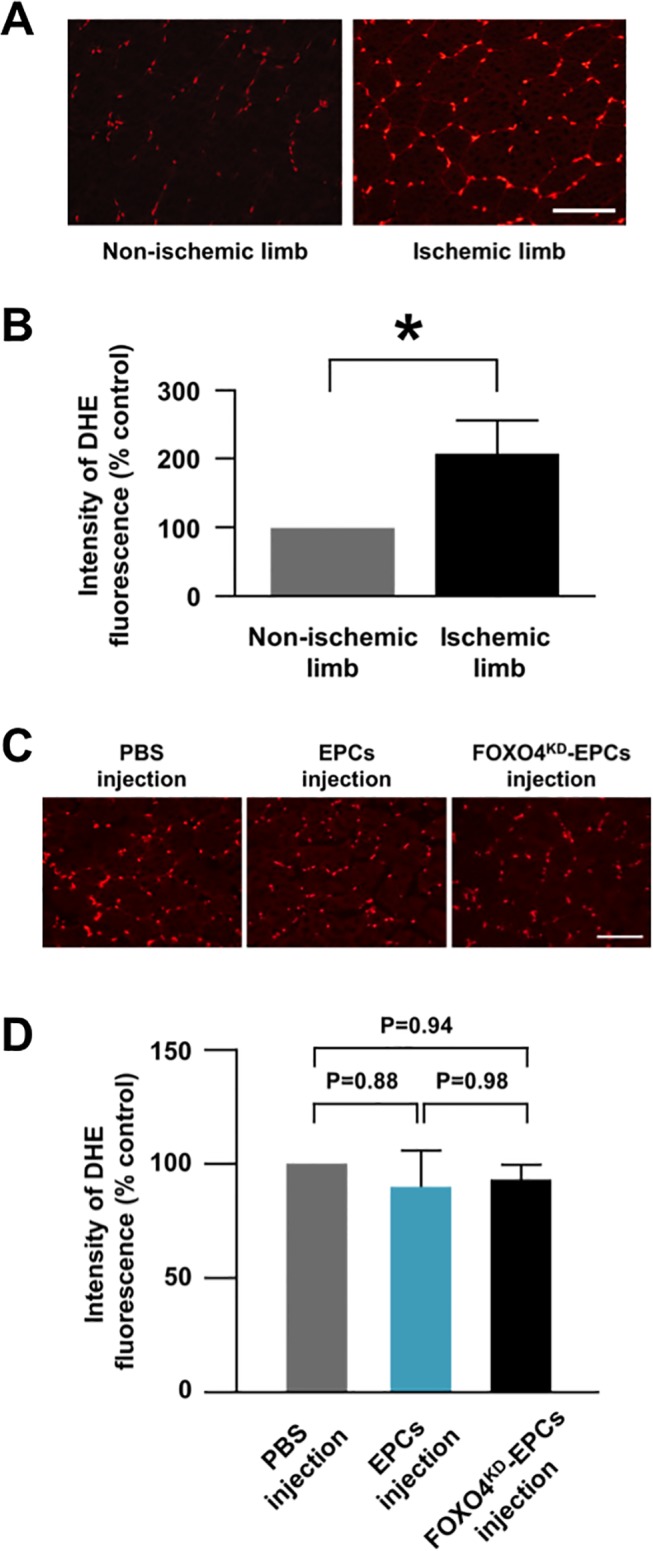
ROS production in athymic nude rat ischemic limbs. (A) Representative fluorescence microscopic images of DHE-stained tissues of the non-ischemic and ischemic limbs of athymic nude rats. DHE was stained red. Scale bar: 100 μm. (B) Pooled data of DHE fluorescence intensity of the rat non-ischemic and ischemic limbs (*: p<0.05; n = 12, each). (C) Representative fluorescence microscopic images of DHE-stained tissues of the ischemic limbs 24 h after intramuscular injection of PBS, EPCs, or FOXO4^KD^-EPCs. Scale bar: 100 μm. (D) Pooled data of DHE fluorescence intensity of the ischemic limbs 24 h after intramuscular injection of PBS, EPCs, or FOXO4^KD^-EPCs (n = 7, each).

## Supporting Information

S1 File Raw data for revised figures (ZIP)
