# Supplementary figures and images for: Correction: FOXO4-Knockdown Suppresses Oxidative Stress-Induced Apoptosis of Early Pro-Angiogenic Cells and Augments Their Neovascularization Capacities in Ischemic Limbs
Source: PLoS One. 2015 Apr 27;10(4):e0127245. doi: 10.1371/journal.pone.0127245 (PMC4411169; doi:10.1371/journal.pone.0127245)

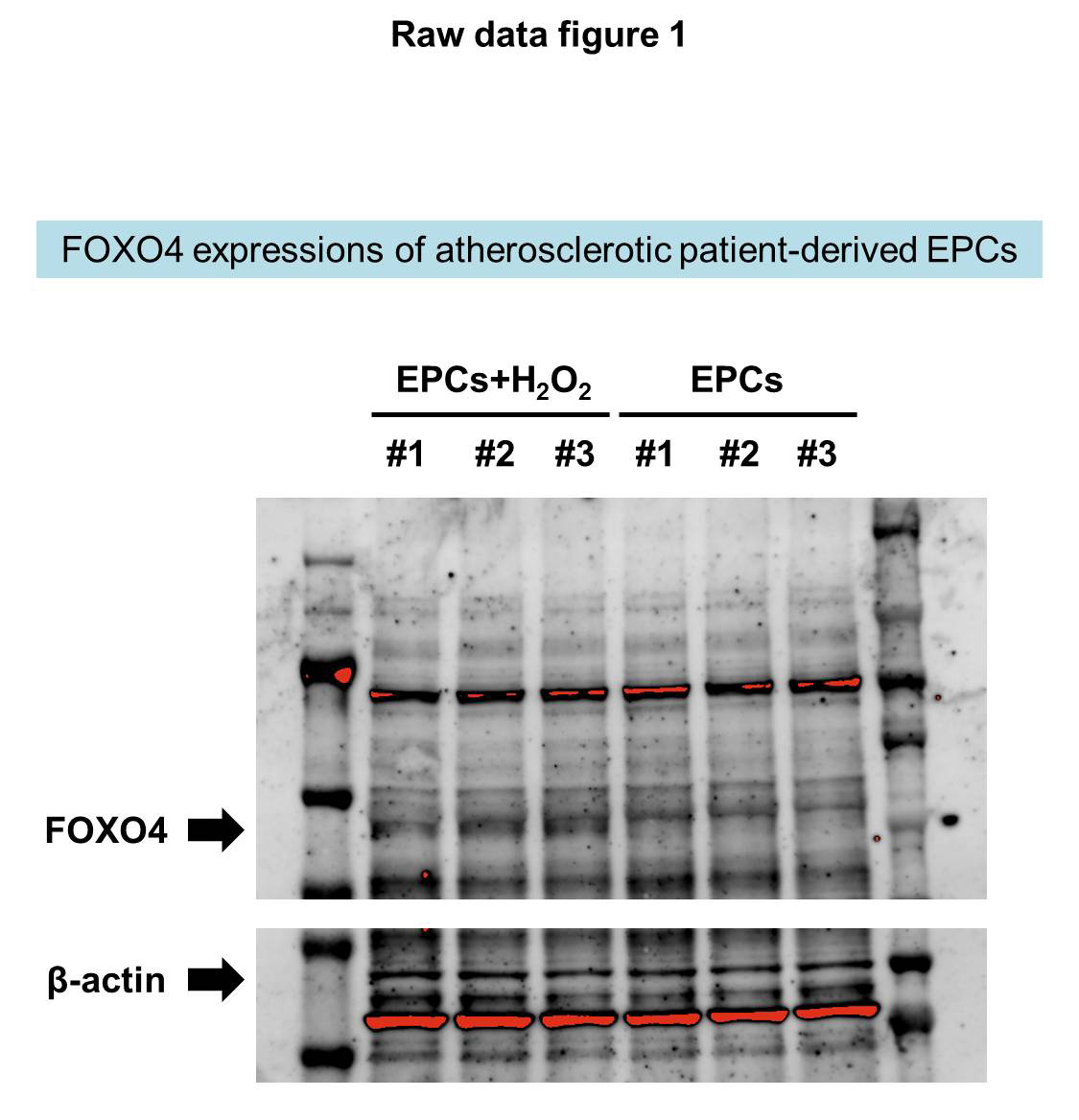

Supplement: S1 File — (ZIP) [file pone.0127245.s001.zip › PONE-D-13-50631 files/Raw data figure 1.jpg]

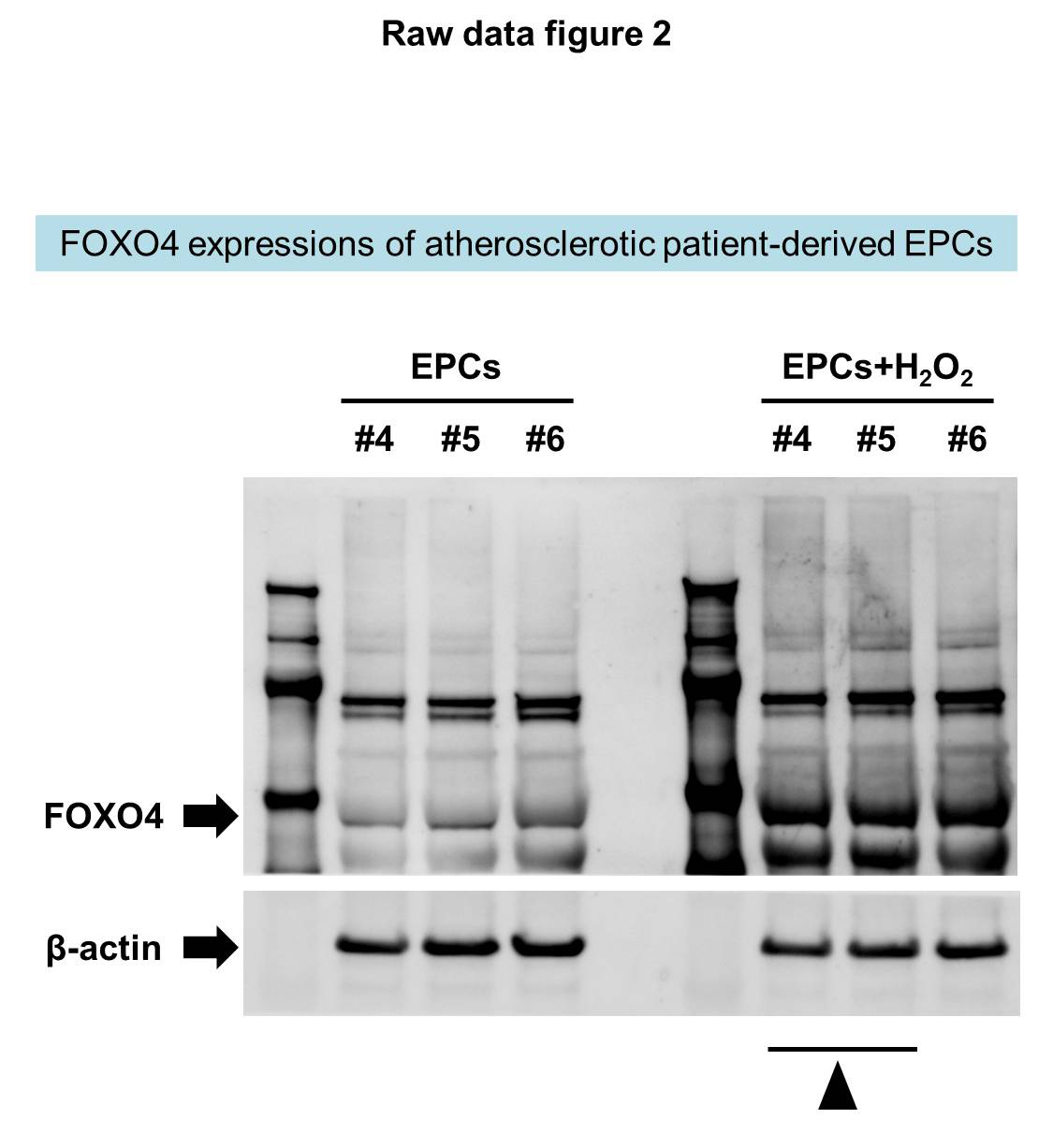

Supplement: S1 File — (ZIP) [file pone.0127245.s001.zip › PONE-D-13-50631 files/Raw data figure 2.jpg]

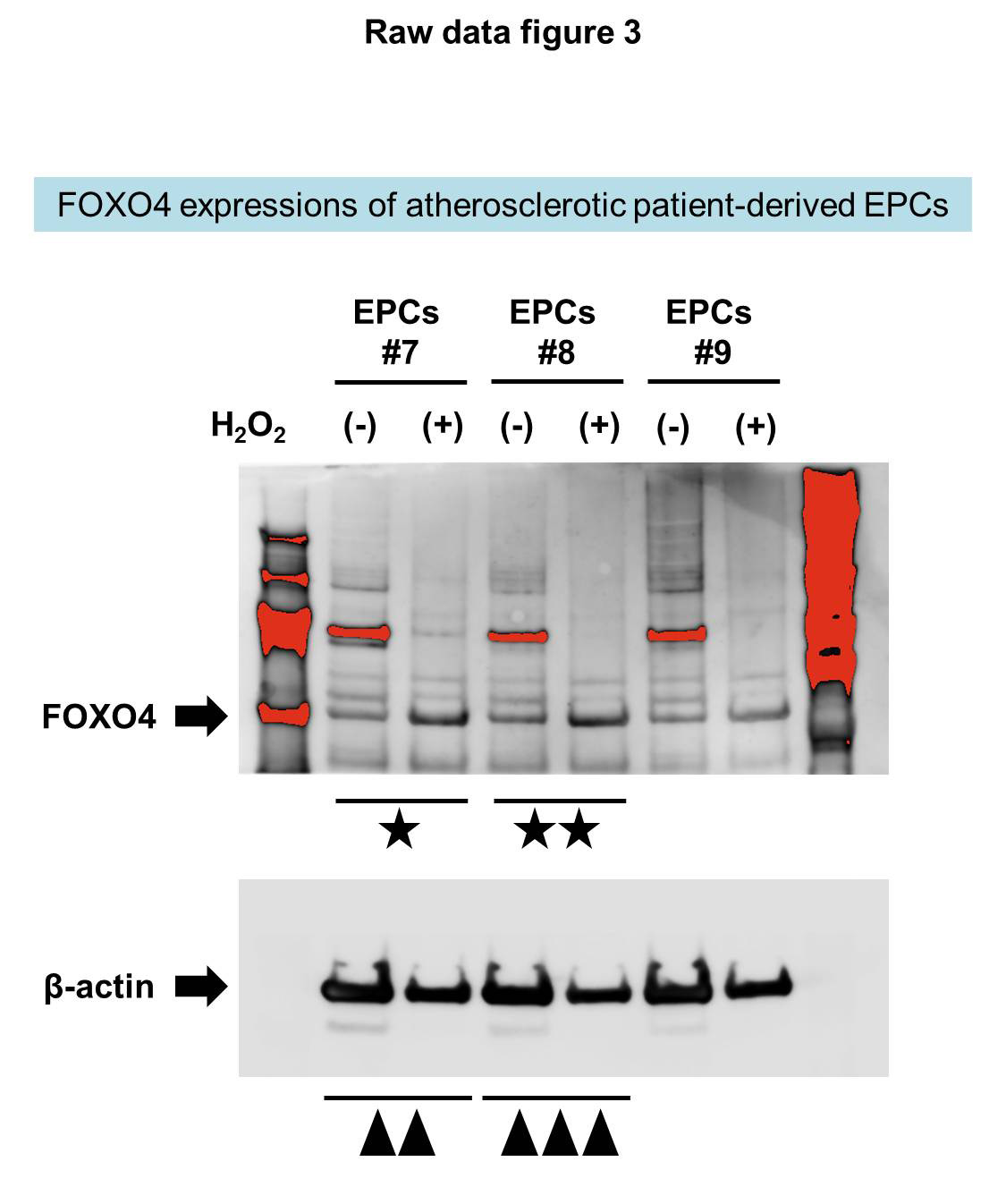

Supplement: S1 File — (ZIP) [file pone.0127245.s001.zip › PONE-D-13-50631 files/Raw data figure 3.jpg]

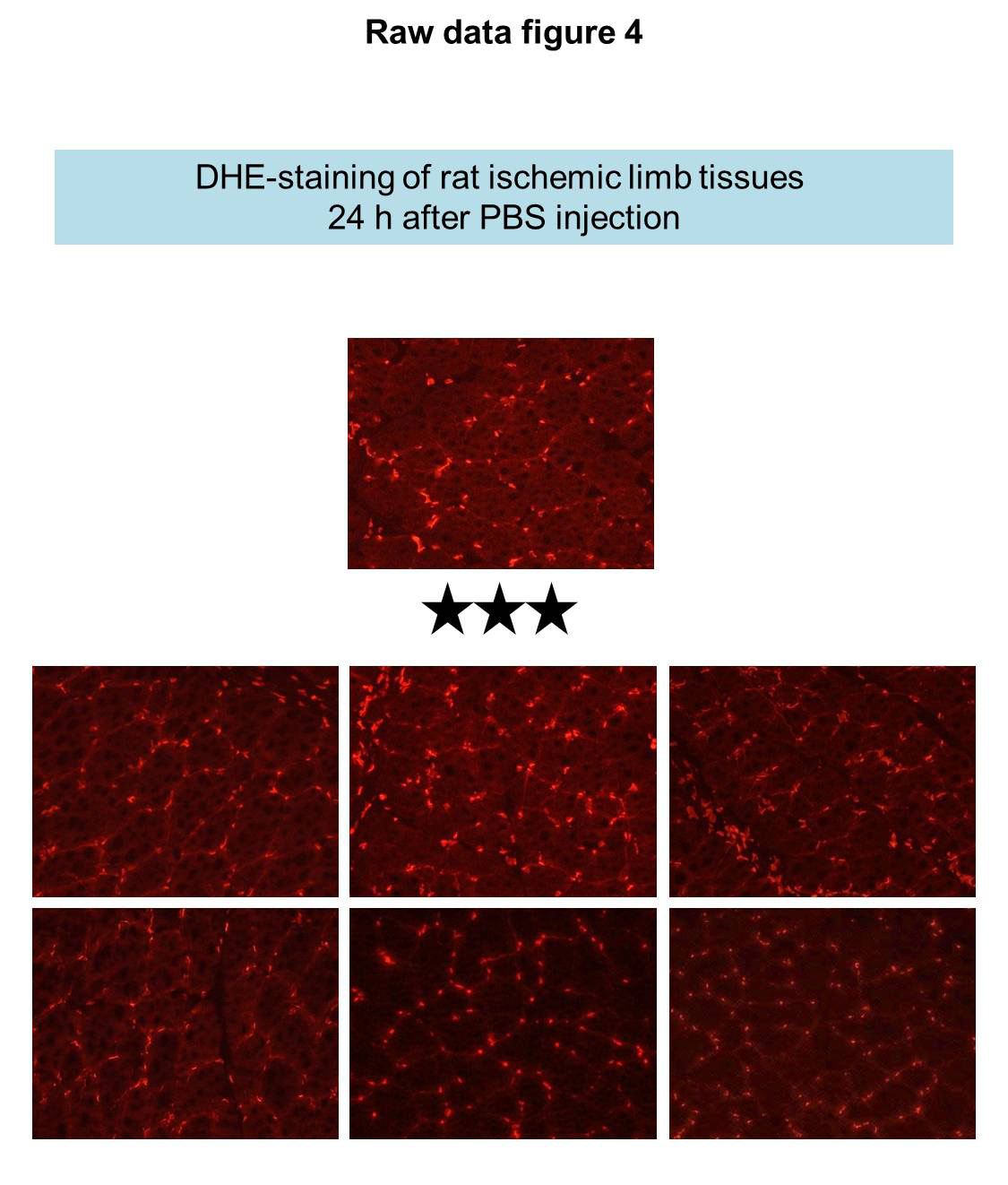

Supplement: S1 File — (ZIP) [file pone.0127245.s001.zip › PONE-D-13-50631 files/Raw data figure 4.jpg]

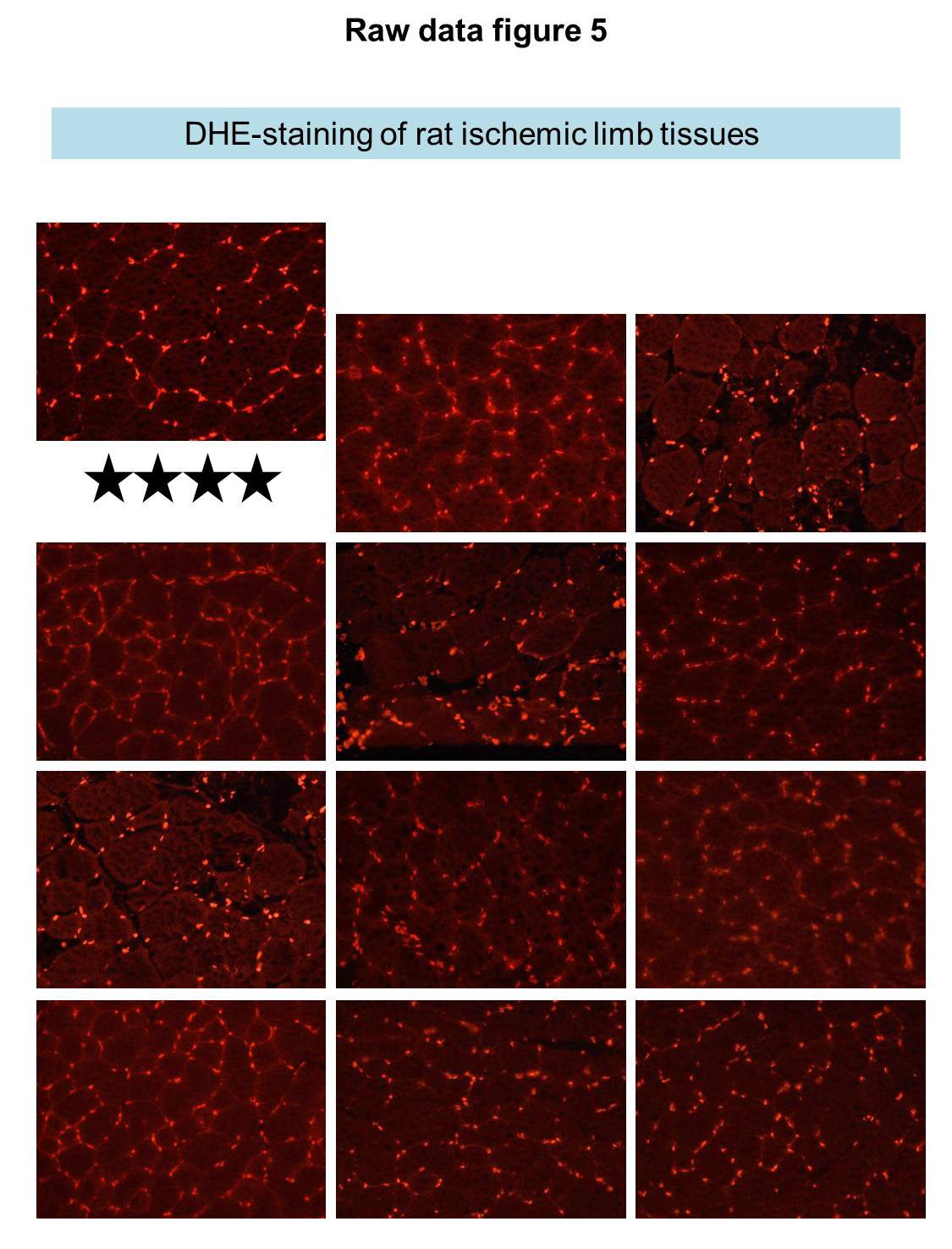

Supplement: S1 File — (ZIP) [file pone.0127245.s001.zip › PONE-D-13-50631 files/Raw data figure 5.jpg]
